# The Life and Death of Jewish Babies

**Published:** 1896-04-04

**Authors:** 


### The Life and Death of Jewish Babies.

Some time ago the members of a committee appointed by charitable Jews to visit and relieve the poor of their nation found with surprise and grief that there were, proportionately to their numbers, far more deaths of children among the Jewish community than among the general population. About 25 per cent. of the deaths in all England are those of children under ten years of age, but in the Jewish community the deaths under that age varied from 54½ in 1891 to 60¼ in 1893. A "Committee on Infant Mortality," to inquire into the causes of this disparity and how far they were preventible, was appointed. Their inquiries were conducted chiefly among the poorest classes, because it is to their ranks that the great majority of these dead children belong. Among the wealthy Jews, indeed, only 20 per cent. of the deaths are those of children; among the middle class 36½; while among the pauper class the deaths of children amount to 81¼ per cent.

This seems to imply, on the face of it, poverty amounting to starvation in the case of certain sections of the Jewish community, if not neglect and cruelty in addition; but statistics are at once the most reliable and the most deceptive things in existence, and when analysed these figures show a state of things, bad enough indeed, but not so bad as they seem. The chief thing to be taken into consideration is the composition of the Jewish community, especially in London. Of late years there has been an immense immigration of foreign, especially Russian and Russo-Polish, Jews into the East-end of London. A fifth of the population of Whitechapel is formed of these, and more than three-fifths of them have come within the last ten years. Now, emigrants are everywhere the cream of population, the men and women in the prime of life. The old people, those over fifty, as a rule remain at home, and take the chances of fate rather than adventure in fresh fields. It is the men and women between twenty and forty who risk the experiment, and try to better their condition in another land. These young men and women sometimes bring young families with them, or have children born to them within the first few years of their settlement in the new country. Of such as these is the Jewish colony in East London largely composed, and there are, therefore, more children in proportion to adults among the Jews than among the general community. Early marriages, and very often imprudent ones, are also common. Statistics show the average age for bridegrooms to be twenty-five, and for brides something under twenty-two. Early marriages mean large families, and, when poverty is added, a great infantile mortality. Poverty usually is added, and with the birth of even the first child charitable aid has to be sought. As, moreover, the birth-rate for Whitechapel, where the foreign Jews mostly congregate, is 40·9 per thousand of the population, while in London generally it is only 31, it may be inferred that large families are commoner among the foreign immigrants than among average Britons.

The death-rate is therefore less alarming than it seems. Where there is a large proportion of children, there will be a large proportion of infant deaths. But even allowing for this, the number of Jewish children who die under the age of ten seems to be cruelly large. Nor can this be wondered at when we realise that all the ills to which the children of the poor are liable exist in an aggravated degree in the homes of these immigrants. These foreign Jews are mostly desperately poor. They do poorly-paid work under the most insanitary conditions. The home is the workshop, which means that children spend their entire lives in one over-crowded and usually ill-ventilated apartment. The trades which these Jews mostly follow, such as tailoring and boot-making, require heating stoves, and are carried on till late at night, with gas flaring for hours. For the stoves coke is often used—a most unhealthy kind of fuel. Ordinary sanitary conveniences are often lacking, and the people have as little notion of cleanliness as of ventilation. They have the prejudice that belongs to all the uneducated against admitting fresh air to a sick room; the risk of cold outweighs, to their mind, the danger of poisoned air. It is rather strange that parents who will let their children play in the street with but the scantiest clothing will exclude the faintest breath of fresh air from their dwellings for fear of cold. There is, moreover, a very general ignorance of the proper diet for children, by no means confined to Jews. "A bit of what's going" is the popular *menu* for children after they leave the breast in all the houses of the working-class, and "what's going" is mostly rough and tough and indigestible; but of 317 deaths investigated by the Jewish Committee, no fewer than 120 were found to be due to diseases of the digestive system—not to starvation, be it understood. Dr. Sequeira especially notes that "a large proportion of the illness among poor children is due to over or improper, and not to under feeding." Thus a large proportion of the deaths were due to causes which are distinctly remediable, though, perhaps, not under existing conditions. Ignorance can only be cured by degrees, but the committee have recommended that a paper containing rules of health for women and young children should be drawn up in both English and Yiddish, and distributed by the clergy and by lady visitors. They are prepared also to call in the assistance of sanitary inspectors and factory officials to prevent rooms which are used as workshops by day being used at night as bed-rooms for children. With regard to the bad nursing which so often brings about fatal results, they wish to see the staff of nurses who work among the Jewish poor increased, and will try to enforce the acceptance of the nurses' aid by refusing charitable relief in cases where these ministrations are declined. If these recommendations can be carried out much will be done to save the lives and preserve the health of Jewish children. And they have, as it is, many things in their favour. The committee did not find in their investigations that Jewish parents let their children die from neglect in order to obtain the money for insurance policies, or got drunk, and overlay them. They did not find illegitimacy and consequent child-murder, nor did they find children whose constitutions had been ruined by the vices of their parents. With wiser feeding and better ventilation, the Jewish baby has, perhaps, a better chance of reaching maturity than the Christian.

